# Retrospective Analysis of 50 Postnatal BVDV Outbreaks in Cattle from Central Argentina: Clinical, Pathological, and Epidemiological Insights

**DOI:** 10.3390/v17101359

**Published:** 2025-10-11

**Authors:** Emiliano Sosa, Evangelina Miqueo, Gina Rustichelli Millán, Maximiliano Spetter, Enrique Louge Uriarte, Juan Livio, Martina Pachiani, Juan Agustín García, Eleonora Morrell, Marisol Yavorsky, Andrea Elizabeth Verna, Erika González Altamiranda, Germán José Cantón

**Affiliations:** 1 Instituto de Innovación para la Producción Agropecuaria y el Desarrollo Sostenible (IPADS), RN 226 km. 73.5, Balcarce 7620, Argentina; sosa.emiliano@inta.gob.ar (E.S.); ginarustichelli@gmail.com (G.R.M.); maxispetter@gmail.com (M.S.); elougeuriarte@gmail.com (E.L.U.); liviojuan@gmail.com (J.L.); martinapachiani@gmail.com (M.P.); garcia.juanagustin@inta.gob.ar (J.A.G.); morrell.eleonora@inta.gob.ar (E.M.); yavorsky.marisol@inta.gob.ar (M.Y.); verna.andrea@inta.gob.ar (A.E.V.); galtamiranda.erika@inta.gob.ar (E.G.A.); 2 Facultad de Ciencias Agrarias, Universidad Nacional de Mar del Plata, Balcarce 7620, Argentina; miqueo.evangelina@inta.gob.ar

**Keywords:** bovine viral diarrhea virus, postnatal infection, mucosal disease, acute infection, pestivirus

## Abstract

Bovine viral diarrhea virus (BVDV) is an important pathogen in cattle and causes considerable economic losses worldwide. In Argentina, where there is no national control program, BVDV remains endemic. In this retrospective study, the epidemiological, clinical and pathological features of postnatal BVDV-associated diseases in 50 outbreaks in central Argentina (1995–2024) were analyzed. Data were obtained from field reports, necropsies, and virological results (virus isolation, RT-nPCR, immunochromatography). No seasonal pattern was found. Acute infections (AIs) and mucosal disease (MD) occurred with similar frequency. Clinical signs included salivation, weakness, emaciation and diarrhea. The lesions were widespread and involved the gastrointestinal tract, skin, lymphoid tissues and spleen. Although MD cases has more extensive tissue involvement, no significant differences in morbidity, mortality or distribution of lesions were observed between AIs and MD. BVDV-1b was the most frequently detected subtype. These results highlight the challenges of BVDV control in extensive production systems. Strengthening diagnostic surveillance, implementing targeted vaccination and eliminating persistently infected animals are essential to reduce BVDV impact in endemic regions such as Argentina.

## 1. Introduction

Bovine viral diarrhea virus (BVDV) is an important pathogen in the livestock industry and causes significant economic losses worldwide due to infections in cattle and contamination of biological products [1]. It is the main member of the genus *Pestivirus* within the family *Flaviviridae*, which comprises three recognized species: *Pestivirus bovis* (BVDV-1, with at least 22 subtypes), *Pestivirus tauris* (BVDV-2) and *Pestivirus brazilense* (BVDV-3, or HoBi-like pestivirus). Both BVDV-2 and HoBi-like pestiviruses are classified into four subtypes [2]. Finally, BVDV strains are categorized into two biotypes based on their effect on cell cultures: cytopathic (cp) and non-cytopathic (ncp) [1].

Cattle can be infected with BVDV at any age, from the embryonic period to adulthood. Clinical manifestations range from subclinical infections to severe consequences such as abortion, mucosal disease (MD), and hemorrhagic syndrome [1,3]. In addition, the ncp-BVDV biotype can cross the placenta during early pregnancy, resulting in the birth of clinically healthy and persistently infected (PI) calves. These PI animals play a central role in the epidemiology of the disease, as they constantly shed BVDV and serve as the primary source of infection within herds. Consequently, the identification and removal of PI animals is an essential component of BVDV control strategies [4]. In contrast, postnatal acute BVDV infections (AI) usually result in mild clinical signs and short-term virus shedding, representing a lower risk of transmission [5]. Several factors such as management practices, the introduction of purchased animals, direct contact with neighboring farms, herd size and the presence of PI cattle have been associated with an increased prevalence of BVDV-related disease [6]. Effective control and eventual eradication of BVDV requires accurate diagnostic methods, implementation of appropriate vaccination protocols and the removal of PI animals from herds [7,8]. However, the sensitivity and specificity of available diagnostic methods vary, so a combination of several methods is required to improve detection accuracy [8]. Vaccination with homologous circulating strains remains one of the most important strategies for prophylaxis, although the emergence of new BVDV strains and variants may affect the efficacy of vaccination [7].

Despite the implementation of eradication programs in many countries, which are primarily based on the detection and culling of PI cattle, BVDV continues to have a high prevalence worldwide [3]. Middle- to low-income countries tend to have higher BVDV seroprevalence rates than high-income regions, with an average seroprevalence of 45% reported in South America [9]. In Argentina, reported seroprevalence in cattle ranges from 37% to 100% [10,11], with un-vaccinated animals having an estimated exposure rate of around 70% [12]. Global pooled estimates of PI prevalence at the animal level vary by region and are categorized as low (≤0.8% in Europe, North America, and Australia), moderate (>0.8 to 1.6% in East Asia) and high (>1.6% in West Asia) [13]. In Argentina, BVDV was detected in 1% of cattle and 20% of herds [14]. In the central region of the country, BVDV was identified as the cause of abortions in 2.2% of bovine fetuses tested, leading to estimated economic losses of almost USD 4 million in the beef industry and USD 2 million in the dairy industry [15]. In addition, BVDV-1b was the most frequently detected subtype in aborted fetuses, while neutralizing antibodies against BVDV-1a, BVDV-1b, and BVDV-2b were present in most of the fetuses examined [16]. Postnatal BVDV infections can be associated with short-term leukopenia, lymphopenia and/or thrombocytopenia, thymic apoptosis, pyrexia, and diarrhea. The resulting immunosuppression can in turn favor infection with other pathogens or the recurrence of latent bacterial infections [17].

PI cattle can serve as a potential source of emerging viral mutants generated by RNA recombination [18]. In this context, MD is typically associated with reinfection by a cp-BVDV strain that either arises by mutation from the ncp strain already present in the PI animal or is acquired from another PI animal with a homologous cp-BVDV isolate [17]. MD usually affects cattle between 6 and 24 months of age and is characterized by a high mortality rate, with affected animals usually dying within one to two weeks of the onset of clinical signs [19]. This fatal form of the disease is characterized by erosions and ulcerations of the gastrointestinal tract and skin [17,20]. Microscopically, lymphoid depletion, necrosis and vacuolization of epithelial cells in the gastrointestinal tract are frequently observed [19]. However, the clinical and pathological differentiation between AI and MD is often difficult. In this context, cp-BVDV strains are rare and primarily involved in MD outbreaks, whereas ncp strains are more common in nature and are often associated with the most severe forms of AI [21]. In summary, BVDV infection can lead to a wide range of clinical outcomes that are influenced by host-specific factors, environmental conditions and viral characteristics [22].

The aim of this study was to analyze BVDV-associated postnatal outbreaks in cattle in central Argentina through a comprehensive assessment of epidemiological data, clinical presentations and pathological findings, and to highlight common patterns and disease characteristics.

## 2. Materials and Methods

### 2.1. Study Design and Sample Origin

This retrospective study is based on a comprehensive database of all postnatal BVDV outbreaks recorded and confirmed by the Specialized Veterinary Diagnostic Service of INTA Balcarce over a 30-year period (1995–2024), particularly in the central region of Argentina. The database was created from clinical, pathological and epidemiological records documented in real time by our team during each outbreak. Although this is a retrospective study, the data does not originate from secondary sources but from prospectively collected field cases. All included outbreaks met strict inclusion criteria: compatible clinical signs, characteristic gross and histopathological lesions and laboratory evidence of BVDV infection by virus isolation, RT-nPCR or lateral immunochromatography. In this study, cases were categorized as acute infection (AI) or mucosal disease (MD), based on the biotype of the isolated virus (ncp or cp) according to established literature criteria [21].

### 2.2. Diagnostic Criteria and Case Classification

The diagnostic criteria were based on anamnesis, clinical signs, compatible gross and histological lesions and laboratory detection of BVDV using various virological methods. Epidemiological data were collected for each outbreak, including geographical location, date (month–year), production system, age of affected animals, clinical presentation (AI or MD), and morbidity, mortality and lethality rates. A total of 64 autopsies were performed and the most important gross pathological findings were documented. Tissue and blood samples were taken for virological analysis. Virus isolation (VI) and nested RT-PCR (RT-nPCR) were performed on spleen, whole blood and serum samples according to the methodology described by Spetter et al. [16]. In addition, ear biopsies were tested for BVDV using lateral immunochromatography (LI) (IDEXX BVDV Antigen Test, IDEXX Laboratories, Westbrook, Maine, USA). Cases were classified as MD if cp-BVDV was isolated and as AI if only ncp-BVDV was isolated. This classification was based on criteria previously described in the literature [5,17,19].

### 2.3. Histopathology

Tissue samples of lung, encephalon (including cerebrum, cerebellum, and medulla oblongata), lymph nodes, heart, thymus, kidney, liver, skeletal muscle, hard and soft palate, tongue, esophagus, reticulum, rumen, omasum, abomasum, small and large intestines, spleen, adrenal glands, skin, and thyroid gland were routinely collected and fixed in 10% buffered formalin (pH 7.2) for 72 h by our diagnostic team. The samples were then processed, sectioned at 4.0 µm, and stained with hematoxylin and eosin (HE).

### 2.4. Statistical Analysis

All statistical analyses were performed with RStudio (R version 4.4.0, 2024). The distribution of BVDV outbreaks across seasons and production systems was assessed using the Chi-squared goodness-of-fit test. Due to low frequencies in some categories, production systems were summarized into two broader groups: ‘beef systems’ and ‘dairy systems’.

Clinical presentations (AI or MD) were assessed using a Chi-squared test for goodness-of-fit. Comparisons were made using the non-parametric Wilcoxon rank-sum test to assess differences in morbidity, mortality and lethality rates.

Linear models with morbidity, mortality and lethality as dependent variables were created to assess the impact of vaccination history on health outcomes. Calves and dams were categorized according to their vaccination history, with categories defined as ≤1 dose (no or a single vaccination) or ≥2 doses (two or more vaccinations). The assumptions of the model were tested for normality of the residuals using the Shapiro–Wilk test and for homoscedasticity using the Breusch–Pagan test. If the residuals deviated from normality, logarithmic transformations of the response variables were performed. Pairwise comparisons between vaccination categories were performed using estimated marginal means (emmeans) with Tukey adjustment.

The distribution of clinical signs was analyzed using Fisher’s exact test applied to both the full contingency table and grouped clinical signs categorized by organ systems (systemic, digestive, respiratory, neurological and other). The association between disease presentation (AI vs. MD) and age category was assessed using ordinal logistic regression, with statistical significance determined using Wald tests.

Associations between BVDV type/subtype and clinical presentation (AI or MD) were assessed using contingency tables. Due to the small expected number, Fisher’s exact test was used. Analyses were performed separately by type and subtype.

The gross and microscopic lesions were analyzed using frequency tables. The presence of individual lesions was compared between the AI and MD groups using Fisher’s exact test. To compare the number of affected tissues per animal, the Shapiro–Wilk test was first applied to assess normality. If the data were not normally distributed, the Wilcoxon rank-sum test was used; otherwise, Welch’s t-test was used to compare normally distributed variables, such as the number of affected organs.

To investigate tissue and organ involvement per protocol, the number of affected tissues was compared between groups using the Wilcoxon rank-sum test, as the distribution was not normal, while the number of affected organs followed a normal distribution and was analyzed using Welch’s *t*-test.

For lesion-specific comparisons, contingency tables were created for each organ and lesion type to assess their association with clinical presentation. For proportional comparisons, Fisher’s exact test was used, with Monte Carlo simulation performed for tables with multiple categories or low expected cell counts. Chi-squared tests were used when the assumptions for larger contingency tables were met.

In addition, 2 × 2 tables were created for each lesion type to compare the presence or absence of the lesion between AI and MD cases. Fisher’s exact test was used to determine *p*-values and odds ratios. Lesions with low frequency or incomplete tables were excluded from this analysis. Results were sorted by *p*-value and the clinical presentation in which each lesion was observed more frequently was indicated. A significance level of *p* < 0.05 was used for all analyses.

## 3. Results

### 3.1. Epidemiological Data

Clinical outbreaks associated with BVDV infection occurred throughout the year, with cases reported in summer (32%; 16/50), autumn (24%; 12/50), winter (24%; 12/50), and spring (20%; 10/50); no seasonal pattern was observed (χ^2^ = 1.52, *p* = 0.68). Most BVDV-associated outbreaks occurred in beef cattle (49/50); only one outbreak was recorded in dairy cattle (χ^2^ = 38.72, *p* < 0.01). In beef systems, outbreaks were reported in cow–calf systems (81.63%; 40/49), feedlots (8.16%; 4/49), wintering systems (6.12%; 3/49) and bull breeding farms (4.08%; 2/49). BVDV-infections were detected in cattle younger than 8 months (22.95%; 14/61), between 8 and 12 months (27.87%; 17/61), and older than 12 months (49.18%; 30/61). The distribution of age groups did not differ significantly between AI and MD presentations (χ^2^ = 0.71, *p* = 0.70). In 68% of the outbreaks (34/50), the animals originated from the same farm, while in 26% (13/50) they had been introduced from another farm. In 6% of cases (3/50) no information on the origin was available.

In 58% of outbreaks (29/50) a previous BVDV vaccination had been carried out, while in 20% (10/50) no vaccination history was available; no information was available for 11 outbreaks. Of the vaccinated calves, 3.45% (1/29) received one dose, 79.31% (23/29) received two doses and 17.24% (5/29) received three doses post-weaning. In the cases where information was available, the mothers of the affected calves received one (10.34%; 3/29) or two (27.59%; 8/29) doses of vaccine before breeding, and 6.90% (2/29) received one dose at pregnancy diagnosis (three months after the end of the breeding season). The number of vaccinations in both calves and the vaccination history of their mothers was not significantly related to mortality, morbidity or lethality. In the log-transformed models, vaccination of calves showed no effect on mortality (estimated marginal means: ≤1 = 1.48, ≥2 = 1.52; *p* = 0.95), morbidity (≤1 = 1.11, ≥2 = 1.14; *p* = 0.97), or lethality (≤1 = 85.4, ≥2 = 84.4; *p* = 0.96). Similarly, the birth of calves to vaccinated mothers had no significant effect on mortality (≤1 = 0.96, ≥2 = 2.03; *p* = 0.21), morbidity (≤1 = 0.63, ≥2 = 1.62; *p* = 0.50) or lethality (≤1 = 70.3, ≥2 = 99.5; *p* = 0.38).

On the 50 outbreaks analyzed, 17 (34%) were classified as AI (isolation of ncp-BVDV) and 26 (52%) as MD (isolation of cp-BVDV). In 7 outbreaks (14%), classification was not possible because the BVDV-infection was confirmed by RT-PCR or LI, but the VI test was negative. The distribution of clinical presentations did not differ significantly (χ^2^ = 1.88, *p* = 0.17), indicating an equal distribution between the two clinical forms. No differences were found between genotypes or subtypes in terms of type of presentation (AI Vs. MD) (Fisher’s exact test, *p* = 0.38 and *p* = 0.15, respectively). In AI cases, ncp-BVDV isolates were genotyped as BVDV-1b (64.71%; 11/17), BVDV-2b (11.76%; 2/17), BVDV-2a (5.88%; 1/17) and unidentified (17.65%; 3/17). In the MD cases, only cp-BVDV was isolated in 69.23% of cases (18/26), while co-infections with ncp- and cp-BVDV were detected in 30.77% (8/26) of the MD cases. Isolates from MD cases were classified as BVDV-1b (38.46%; 10/26), BVDV-1a (23.08%; 6/26), BVDV-2b (15.38%; 4/26), BVDV-1i (3.85%; 1/26), and unidentified (19.23%; 5/26). In the remaining unclassified outbreaks, genotyping revealed BVDV-1b in 2 cases, BVDV-1a in 1 case, and unidentified strains in 4 cases. No significant differences were found in average morbidity (AI = 5.25% ± 4.89; MD = 5.88% ± 9.51; *p* = 0.53), mortality (AI = 3.19% ± 3.46; MD = 8.42% ± 8.45; *p* = 0.12) and lethality (AI = 59.31% ± 32.26; MD = 85.6% ± 21.55; *p* = 0.08) between AI and MD presentations. Finally, pharmacotherapy with broad-spectrum antibiotics was administered in 52% of outbreaks (26/50), with no reversal of clinical signs. Supplementary Table S1 summarizes the epidemiological findings of 50 outbreaks caused by BVDV infections.

### 3.2. Clinical Signs

The clinical signs observed in the affected animals were primarily characterized by excessive salivation (52%; 26/50), progressive weakness (52%; 26/50), emaciation (48%; 24/50) and diarrhea (42%; 21/50). In addition, nasal discharge (36%; 18/50), signs of dehydration (30%; 15/50) and fever (26%; 13/50) were frequently observed. Less common signs included dry muzzle (18%; 9/50), dyspnea (18%; 9/50), epiphora (14%; 7/50) and submandibular edema (2%; 1/50). No significant differences were found in clinical signs between AI and MD presentations (Fisher’s exact test, *p* = 0.27). Table 1 shows the frequency of clinical signs observed in AI and MD affected calves.

### 3.3. Gross Findings

Gross lesions were observed in several organs, with no significant overall difference in distribution between AI and MD presentations (Fisher’s exact test, *p* = 0.39). The most commonly affected organs were the esophagus (74%; 37/50), oral mucosa (64%; 32/50), skin (62%; 31/50), abomasum (56%; 28/50), small intestine (48%; 24/50), lymph nodes (38%; 19/50), forestomachs (28%; 14/50), and large intestine (20%; 10/50). Gross lesions did not differ significantly between clinical presentations. However, calves with MD had a significantly higher number of affected organs (mean = 5.7) than those with AI (mean = 3.9; Wilcoxon test, *p* = 0.02). Table 2 provides an overview of the gross lesions observed depending on the type of presentation (AI and MD).

**Figure 1:**
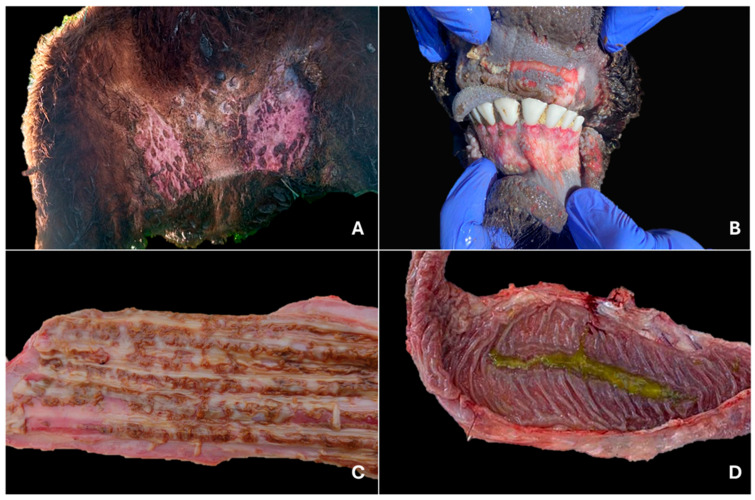
Gross lesions in cattle infected with bovine viral diarrhea virus. (**A**) Bilateral and symmetrical alopecia with severe dermatitis in the inguinal region, characterized by the presence of multiple crusts. Steer, outbreak #45. (**B**) Severe erosive and ulcerative, multifocal to coalescent lesions that show total or partial epithelial loss, as observed in the ventral and dorsal regions, respectively. Calf, outbreak #47. (**C**) Multifocal to coalescent linear erosive lesions with irregular shapes and margins, measuring 1–2 mm in width. Calf, outbreak #49 (**D**) Ileum showing marked congestion, particularly involving Peyer’s patches, with fibrin adherent to the mucosal surface. Steer, outbreak #43.

Among the 64 autopsies performed, skin lesions were observed in half of the cases (50%; 32/64). These lesions were characterized by partially or completely detached black crusts, exposing ulcerated surfaces. They were distributed across various body regions and were occasionally accompanied by fibrinopurulent exudate or myiasis (5/32). Ulcerative dermatitis was most commonly located in the interdigital spaces (40.63%; 13/32), muzzle (28.13%; 9/32), nostrils, and inguinal region (21.88%; 7/32) (Figure 1A). Less frequently, lesions were found in the perineum (15.63%; 5/32), udder, abdominal or neck skin (12.5%; 4/32), axillary region and coronary band (9.38%; 3/32), base of the tail (6.25%; 2/32), dorsum, scrotum, ears, and periocular region (3.13%; 1/32). In two AI cases with ulcerative dermatitis, extensive areas of alopecia were observed on the neck, ventral abdomen, dorsum, inguinal region, and scrotum, characterized by dry, irritated, hyperkeratotic skin with reduced elasticity.

The oral cavity was affected by multifocal ulcerations located on the tongue (46.88%; 30/64), gingiva (40.63%; 26/64), hard palate (31.25%; 20/64), cheeks (21.88%; 14/64), lips (14.06%; 9/64), and soft palate (9.38%; 6/64). Tongues exhibited multiple sharp-edged ulcerations ranging from 1 to 65 mm in length and 1 to 10 mm in width, predominantly located on the lateral surface (70%; 21/30), followed by the ventral and dorsal surfaces (30%; 9/30 each). Similar lesions were observed on both upper and lower gums in most cases (73.08%; 19/26) (Figure 1B), whereas in fewer cases only the lower (15.38%; 4/26) or upper (11.54%; 3/26) gums were affected. Additionally, multifocal ulcerations were occasionally observed in the glottis (4.69%; 3/64) and pharynx (1.56%; 1/64).

The esophagus was affected in most of the autopsied animals (68.75%; 44/64), presenting multiple ulcerations ranging in size from 1 to 20 mm in length and 1 to 10 mm in width (Figure 1C). Occasionally, fibrinous exudate was observed over the ulcerated mucosa. These lesions were most evenly distributed within the esophageal mucosa (79.55%; 35/44), but in some instances, they were limited to the cranial portion (9.09%; 4/44) or the caudal portion near the cardia (9.09%; 4/44).

In the rumen, mucosal erosions with irregular edges measuring 5 to 20 mm in diameter were observed in 25.0% of autopsies (16/64), located on the ruminal pillars in all cases, and in some instances distributed across the entire ruminal mucosa (37.5%; 6/16). These crateriform ulcers reached depths of up to 3 mm.

Less frequently, similar lesions were recorded in the omasum (3.13%; 2/64) and reticulum (1.56%; 1/64). The abomasum was affected in 51.56% of the cases (33/64), with oval to linear ulcers ranging from 1 to 20 mm in diameter, primarily located along the edges of the folds. In one case, abomasal ulcers were observed in the pyloric region, measuring 3 × 5 mm, with irregular edges and a pink base. Occasionally, marked edema of the abomasal mucosa (13.33%; 4/30) and hemorrhagic abomasitis (6.67%; 2/30) were noted in association with the ulcerative lesions.

Lesions in the small intestine were observed in 45.31% of autopsies (29/64). In most cases (88.66%; 26/29), they primarily affected ileum segments, measuring up to 15 × 5 cm, and exhibited a reddish-black discoloration of the intestinal wall and absence of mucosa, which suggested necrosis of Peyer’s patches. In several instances, these areas were covered by fibrinous material (53.85%; 14/26) (Figure 1D) or necrotic exudate (11.54%; 3/26). The remaining cases with intestinal lesions (10.34%; 3/29) were characterized by hemorrhagic enteritis affecting the terminal ileum, with multiple suffusions, associated clots, and hemorrhagic exudate covering the mucosal surface.

Lesions were observed in 18.75% of autopsies (12/64) from the large intestine, which were distributed along the colon (12/12), cecum (6/12), and rectum (5/12). In the colon, pathological changes included linear ulcers measuring 2 to 30 mm in length, 5 to 20 mm in width, and approximately 2 mm in depth (50%; 6/12). Also, hemorrhagic enteritis with mucosal thickening (41.67%; 5/12) and enteritis with hemorrhage and fibrin deposits (8.33%; 1/12) were identified. In the cecum, ulcerative lesions of similar dimensions were observed in most cases (83.33%; 5/6), whereas hemorrhagic lesions with clots adhered to the mucosa were recorded in one case (16.67%; 1/6). In the rectum, ulcerative (40%; 2/5), hemorrhagic (40%; 2/5), and purulent proctitis (20%; 1/5) were identified. Additionally, in eight cases, a pasty black liquid was found at various levels of the gastrointestinal tract, suggesting the presence of digested blood.

Lymphadenomegaly was observed in 29.69% of autopsies (19/64). In all cases, the affected lymph nodes were enlarged and exhibited a yellowish to dark red coloration. The most affected lymph nodes were mesenteric (63.16%; 12/19), followed by retropharyngeal and prescapular (22.32%; 4/19), and less frequently popliteal, subcapsular, and submandibular lymph nodes (5.26%; 1/19).

Pulmonary lesions were found in 23.44% of cases (15/64), mainly in cranial lobes. These included consolidation and emphysema, with affected areas appearing firm and reddish to gray. Liver and heart changes were seen in 7.81% of cases (5/64), including yellowish liver tissue and small hemorrhages on the heart surface.

### 3.4. Microscopic Findings

Histological analysis was performed on tissue samples from 58 autopsies. Microscopic lesions showed a wide range of tissue involvement. No significant differences were found in the overall lesion distribution between AI and MD presentations (Fisher’s exact test, *p* = 0.16), nor in the number of affected tissues per case (*p* = 0.51). Due to the retrospective nature of the study and variability in sample quality, lesion severity was not scored. Future studies may benefit from implementing standardized scoring systems to assess lesion intensity. Table 3 summarizes the microscopic lesions observed according to the type of presentation.

**Figure 2:**
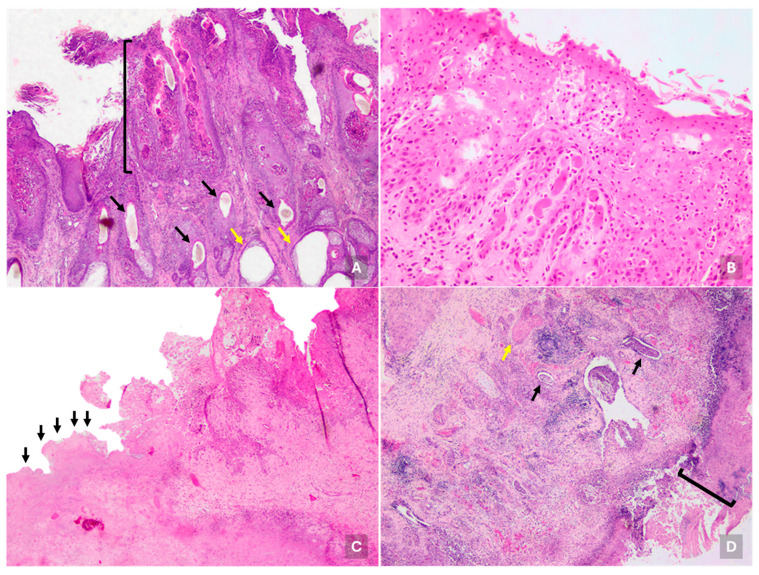
Microscopic lesions in cattle infected with bovine viral diarrhea virus. (**A**) Multifocal-coalescing necrosis with mixed inflammation, fibrinous exudate, and cellular debris (scabs) of the stratum corneum of skin, occasionally affecting stratum spinosum (black bracket). Also, subepithelial multifocal foci of lymphocytes, macrophages, and neutrophils with perivascular distribution were observed. Note the low number of hair follicles (black arrows) and dilation of sweat glands (yellow arrows). H&E, 100×. Steer, outbreak #45. (**B**) Tongue with multifocal erosive lesions characterized by keratinocyte necrosis, predominantly in the stratum corneum and to a lesser extent in the stratum spinosum. H&E, 400×. Calf, outbreak #47. (**C**) Focally extensive area of full necrosis in the esophagus with loss of cellular detail with fibrinous exudate and inflammatory rim between necrotic area (left, black arrows) and more conserved esophagus tissue (right). The more conserved area shows stratum corneum necrosis extending to stratum spinosum, admixed with neutrophils, fibrinous exudate, and cellular debris. H&E, 100×. Calf, outbreak #49. (**D**) Ileum with focal extensive ulceration characterized by expanded mucosa with abundant infiltrate, fibrinous exudate, few crypts remaining with epithelial attenuation, and necrosis and intraluminal cellular debris (black arrows). Also, fibrinous thrombosis is observed (yellow arrow). Villi are absent due to necrosis and replaced by fibrinous exudate, cellular debris, inflammatory cells and superficial bacterial colonies, forming a crust adhered mucosal surface (black bracket). H&E, 100×. Steer, outbreak #43.

Histologic skin lesions were observed in 29.31% of cases (17/58). At the epidermal level, more than half (52.94%; 9/17) showed degeneration and necrosis of the stratum corneum, distributed either multifocally (35.29%; 6/17) or focally (29.41%; 5/17), accompanied by inflammation with polymorphonuclear cells, blood, fibrinous exudate, and cellular debris (scabs) (Figure 2A). In most cases (70.59%; 12/17), the dermis presented multiple perivascular inflammatory foci composed of lymphocytes, macrophages, and neutrophils. Additional findings included absence or atrophy of hair follicles (23.53%; 4/17), keratosis (11.76%; 2/17), dilation of sweat (11.76%; 2/17) and sebaceous (5.88%; 1/17) glands.

Microscopic lesions in the tongue were observed in 41.38% of cases (24/58), characterized by focal loss of epithelial continuity with erosive (28.83%; 5/24) or ulcerative (75%; 18/24) changes, along with epithelial and/or subepithelial necrosis and partial or complete detachment of the epithelium (Figure 2B). In 62.5% of cases (15/24), a subepithelial inflammatory infiltrate composed of lymphocytes, macrophages, plasma cells, and neutrophils was present, often diffusely distributed, and perivascular in some cases (20.0%; 3/15). Cheek samples (*n* = 4) showed similar lesions to those found in the tongue. In one case, the larynx and glottis exhibited extensive epithelial erosion with an inflammatory response dominated by macrophages and lymphocytes, extending into the perivascular area.

At the esophagus, lesions were observed in 36.21% of cases (21/58), affecting the entire wall in a focally diffuse pattern. These lesions included erosions with partial mucosal loss and necrotic debris (33.33%; 7/21), and ulcers with complete epithelial detachment and lamina propria involvement (66.67%; 14/21). In most cases (80.95%; 17/21), these lesions were accompanied by submucosal esophagitis, characterized by infiltration of lymphocytes, macrophages, plasma cells, and neutrophils (Figure 2C). Additionally, hemorrhages were occasionally observed in the submucosa (14.29%; 3/21) and serosa (4.76%; 1/21).

Ruminal lesions were observed in 32.76% of cases (19/58), mainly as diffuse, severe, non-suppurative ruminitis. Most were erosive (73.68%; 14/19), with necrosis of the spinous and corneal epithelium and multifocal-coalescent pustules. The remaining cases showed ulcerative lesions (26.32%; 5/19) with complete epithelial loss and abundant degenerated cells. These lesions were often accompanied by inflammatory infiltrates (63.16%; 12/19) of lymphocytes, macrophages, neutrophils, plasma cells, mast cells, and fibroblasts. In three ulcerative cases, severe mucosal and submucosal hemorrhage was present. Lesions in the reticulum (3.45%; 2/58) and omasum (1.72%; 1/58) were erosive, with epithelial necrosis and multifocal-coalescent pustules. A lymphohistiocytic infiltrate was observed in the lamina propria, along with necrotic debris and fibrin in the lumen.

Abomasal lesions were found in 39.66% of cases (23/58). Ulcerative abomasitis with diffuse epithelial necrosis was present in 34.8% (8/23) of the cases, and erosive lesions with severe hemorrhage and intravascular thrombi in one case (4.4%; 1/23). Most cases (91.30%; 21/23) showed submucosal infiltrates of lymphocytes, macrophages, and neutrophils. Ulcerative necrosis was associated with glandular destruction and intraluminal necrotic content, and in one case, abundant intralesional bacteria. These lesions were sometimes accompanied by hemorrhage (43.5%; 10/23) and edema (17.4%; 4/23) in the mucosa and submucosa.

Lesions in the small intestine were highly prevalent, affecting 79.31% of cases (46/58), predominantly in the ileum (93.5%; 43/46), and less frequently in the jejunum (4.4%; 2/46) and duodenum (2.2%; 1/46). A common finding was the destruction of the epithelial lining of the crypts of Lieberkühn (56.5%; 26/46), often accompanied by marked villous atrophy (26.1%; 12/46). More than half of the affected cases (56.5%; 26/46) showed mucosal necrosis, either erosive (42.3%; 11/26), involving the crypt epithelium, or ulcerative (57.7%; 15/26), with deeper necrosis extending into the submucosa (Figure 2D). Inflammatory infiltrates were present in 84.78% of cases (39/46), affecting mucosa, submucosa, and muscular layers. These were mainly composed of lymphocytes, macrophages, and plasma cells (79.49%; 31/39), and in some cases neutrophils (20.51%; 8/39), forming microabscesses in crypts (7.69%; 3/39) and interglandular submucosa (2.56%; 1/39).

Dilation of the crypts of Lieberkühn was frequently observed (63.04%; 29/46), with intraluminal accumulation of necrotic cells, inflammatory cells, and mucus. In some cases (20.7%; 6/29), this material extended into submucosa. Detachment of the mucosa with cellular debris, inflammatory cells, and fibrin forming pseudomembranes was noted in 8.69% of cases (4/46). At the level of Peyer’s patches, necrosis and lymphocyte depletion were observed in 21.7% of cases (10/46). One case presented abscesses with a caseous eosinophilic center, surrounded by inflammatory cells and a thin fibroblastic capsule. Finally, severe hemorrhage in the mucosa and submucosa was recorded in 19.6% of cases (9/46).

Lesions in the large intestine were observed in 25.86% of cases (15/58), mainly affecting the colon (86.68%; 13/15), and occasionally the cecum (6.67%; 1/15) and rectum (6.67%; 1/15). Most colon lesions were erosive (76.92%; 10/13), while three cases presented ulcerations. Inflammatory changes in the mucosa and submucosa were common (61.54%; 8/13), with infiltrates composed of lymphocytes, macrophages, plasma cells, and eosinophils. Crypt dilation was noted in nearly half of the cases (46.15%; 6/13), often containing necrotic debris and mucus. In some instances (23.08%; 3/13), crypts extended into the submucosa. One case showed a prominent pseudomembrane in the colonic lumen, composed of inflammatory cells, fibrin, cellular debris, and bacterial colonies. Hemorrhage (30.77%; 4/13) and edema (23.08%; 3/13) were also observed in mucosa and submucosa.

The cecum presented erosive lesions, dilated crypts with necrotic material, and a dense inflammatory infiltrate in the mucosa and submucosa, predominantly lymphocytic and with a clear perivascular distribution. In the rectum, glandular dilation and necrosis were accompanied by hemorrhagic areas and lymphocytic inflammation.

The spleen was affected in 46.55% of cases (27/58), with most showing evident lymphoid depletion (77.78%; 21/27), sometimes accompanied by lymphoid necrosis with karyolitic and karyorrhectic cells (38.10%; 8/21). Splenitis was observed in 22.22% of cases (6/27), characterized by neutrophilic infiltrates surrounding the white pulp (66.67%; 4/6) and lymphohistiocytic infiltrates in the splenic serosa (33.33%; 2/6). Additionally, hemosiderin accumulation (22.22%; 6/27) and severe hemorrhage (11.11%; 3/27) were noted.

Lymph node alterations were present in 41.38% of cases (24/58), including lymphocytic depletion and necrosis in the cortex (33.33%; 8/24), paracortex (8.33%; 2/24), and cortico-medullary region (4.17%; 1/24). Adenitis was frequent (62.5%; 15/24), with macrophage and neutrophil infiltrates located in the cortex (60.0%; 9/15), cortico-medullary region (20.0%; 3/15), and medulla (20.0%; 3/15). Severe hemorrhages were observed in five cases, affecting both cortex and medulla.

Histopathological changes in the liver were observed in 62.07% of cases (36/58). The most frequent findings included lymphohistiocytic infiltrates in the portal spaces (52.8%; 19/36) and centrilobular lipid degeneration (58.33%; 21/36). Less commonly, pigment retention (13.9%; 5/36) and generalized hemorrhages (2.8%; 1/36) were also noted.

The lungs were affected in 50.0% of cases (29/58), primarily due to interstitial pneumonia with lymphocytic and macrophagic infiltrates (79.31%; 23/29), and widespread emphysema (55.17%; 16/29). Bronchopneumonia was observed in a few cases (13.79%; 4/29), characterized by mixed inflammatory infiltrates (lymphocytes, macrophages, neutrophils) and necrotic exudate. Additionally, generalized edema (13.79%; 4/29) and hemorrhages (10.34%; 3/29) were sporadically noted.

Kidney lesions were observed in 32.76% of cases (19/58), characterized by lymphohistiocytic nephritis in the renal cortex. In a few cases (10.53%; 2/19), proteinaceous material was noted within renal tubules. The central nervous system was affected in 18.97% of cases (11/58), with predominant findings of multifocal perivascular gliosis (90.91%; 10/11), lymphohistiocytic meningitis (18.18%; 2/11), and Purkinje cell dysgenesis in the cerebellum (9.09%; 1/11). Cardiac lesions were identified in 10.34% of cases (6/58), consisting of lymphohistiocytic infiltrates located in the endocardium (16.67%; 1/6), myocardium (66.67%; 4/6), and epicardium (33.33%; 2/6). Finally, adrenal gland alterations were observed in 3.45% of cases (2/58), including lymphoplasmacytic infiltrates in the medulla (50.0%; 1/2) and serosa (50.0%; 1/2), as well as blackish pigmentation due to melanin accumulation (50.0%; 1/2).

### 3.5. Diagnostic Techniques

In all analyzed cases, BVDV infection was confirmed using at least one of the diagnostic techniques mentioned above. In most outbreaks, BVDV was isolated (86%; 43/50), with cp-BVDV detected in 41.86% (18/43), ncp-BVDV in 39.53% (17/43), and both cp- and ncp-BVDV in 18.60% (8/43) cases. RT-nPCR was positive in the majority of outbreaks (84%; 42/50). BVDV type 1 was confirmed in 64% (32/50) of cases, type 2 in 14% (7/50), and undetermined types in 22% (11/50). Regarding subtypes, 1b was detected in 46% (23/50), 1a in 14% (7/50), 2b in 12% (6/50), 2a and 1i each in 2% (1/50), and undetermined subtypes in 24% (12/50). Finally, LI was positive in 18% of cases (9/50), confirming the presence of BVDV on skin biopsies. In seven outbreaks, VI was unsuccessful, preventing type and subtype identification. Table 4 presents the results of various diagnostic techniques applied to the 50 outbreaks caused by BVDV infection.

## 4. Discussion

This study describes the epidemiological, clinical, and pathological findings in 50 outbreaks of postnatal BVDV-associated disease in cattle occurring over a 30-year period in Central Argentina. Despite awareness of the disease among farmers and veterinary practitioners, BVDV outbreaks have been occurring consistently over time, highlighting the need to strengthen control measures.

In the present study, we also aimed to compare the different epidemiological, clinical, and pathological aspects between AI and MD. However, it is recognized that sampling limited to certain organs might not reflect the complete distribution of the virus in the animal. Therefore, the possibility of a heterogeneous distribution of cp and ncp BVDV strains in infected animals cannot be ruled out, and the presence of cp strains in unsampled organs remains a possibility. For future investigations, it is recommended to expand sampling to multiple organs in order to improve the accuracy of clinical classification. Additionally, in seven outbreaks, VI was unsuccessful despite repeated attempts. This failure may be attributed to poor sample quality, low viral load, contamination, or other interfering factors that could have hindered viral replication in cell culture systems [17].

Countries that have not implemented BVDV official control programs, including vaccination strategies, tend to exhibit the highest prevalence of PI animals [13]. In Argentina, there are currently no official BVDV control programs in place. Inactivated vaccines are commercially available, typically in combination with other antigens. These vaccines are safe but require booster doses to maintain protective antibody levels [23]. However, most vaccines approved by the National Health Service and Food Quality (SENASA) contain only BVDV-1a, and occasionally BVDV-2 [24]. In the present study, no significant differences were observed in morbidity, mortality or lethality rates between animals with a history of vaccination and those without. This lack of difference may be attributed to several factors, including the small sample size, incomplete vaccination protocols, and the limited antigenic coverage of commercially available vaccines. It was noted that not all animals had been vaccinated, or only a single dose had been administered. This, combined with the absence of coverage for specific subtypes, may have resulted in inadequate protection. Furthermore, the high heterogeneity among viral strains and the uncertain antibody titers required to confer protection against BVDV infection underscore the need to expand control measures [25].

As demonstrated in this study, there was considerable variability in the BVDV subtypes detected across outbreaks. In Argentina, previous studies have also reported a higher prevalence of BVDV subtype 1b compared to others [24], which is consistent with our findings. This trend also aligns with global reports, where the number of viral isolates described for BVDV-1 is considerably higher than for BVDV-2. Among BVDV-1 subtypes, 1b is the most frequently reported worldwide, followed by 1a and 1c [26]. Given the widespread circulation of certain subtypes (particularly BVDV-1b) and their association with both acute and persistent infections, one of the cornerstones of successful disease control is the early detection and elimination of PI animals, which serve as the main source of viral dissemination within herds [27]. Argentina’s most common beef cattle production systems operate under extensive conditions, making the implementation of disease control measures, particularly the identification and removal of PI animals, especially challenging. In addition, interspecies transmission involving other ruminants represents a significant epidemiological factor that can hinder control efforts [20,28]. This aspect is especially relevant given that sheep and goat farming is common in Central Argentina and often overlaps geographically with cattle production [29]. Moreover, recent evidence of BVDV RNA detection in free-living wild boars in Brazil highlights the potential role of wildlife as a reservoir, particularly in extensive livestock systems where contact with wild species is more likely [30]. This underscores the need to consider wildlife in the design and implementation of comprehensive BVDV control strategies in Argentina.

Although most of the outbreaks analyzed in this study occurred in beef production systems, this may be partially explained by the fact that INTA Balcarce is in a region where the proportion of beef cattle farms is significantly higher than that of dairy farms [31]. In this context, most of the outbreaks were recorded in cow–calf systems. It is important to note that the production system in which the outbreak originated does not necessarily correspond to the age of the affected animals at the time of diagnosis. In fact, clinical cases were detected in animals of various ages, including those older than 12 months, indicating that many animals remain on the same farm throughout different production stages. This dynamic may lead to an apparent mismatch between the production system and the age of affected animals, which should be considered when interpreting epidemiological data. Therefore, outbreak characterization must consider both the production context and the individual trajectory of animals within the system. Clinical and pathological findings were consistent with those previously reported [5,17,19,31], which include weight loss, diarrhea, dehydration, and multiple erosions and ulcerations in the gastrointestinal tract. In the analyzed cases, lesions were predominantly located in the intestine, particularly in the ileum. This may be related to findings from certain studies in which BVDV viral loads were highest and most uniformly distributed in the ileum [32]. In addition to intestinal lesions, this viral agent has also been commonly associated with ulcerative lesions in the skin and upper digestive tract [33]. It has been proposed that activation of the intrinsic apoptosis pathway is a key element in the pathogenesis of MD lesions in PI cattle infected with BVDV [34]. Although MD involved a greater number of affected tissues, no significant differences were found in morbidity, mortality, lethality, or lesion distribution. These results suggest that clinical presentation does not always correlate with pathological severity. Although the differences were not statistically significant, the absolute values suggest a trend toward higher lethality in MD, consistent with clinical descriptions in the literature [19]. The absence of significant differences between AI and MD may be attributed to high intra-group variability and a limited sample size. Additionally, the diagnostic service is often called upon in cases involving high mortality, which may introduce bias in the interpretation of epidemiological data, not reflecting the reality of the disease in cases with lower incidence. Therefore, the indices of AI and MD may not necessarily be comparable or reflect similar patterns. Moreover, BVDV-induced immunosuppression may predispose animals to secondary infections, potentially exacerbating clinical signs and explaining the occurrence and severity of many observed lesions [1,17]. These coinfections pose a significant threat to the livestock industry, complicating both outbreak control and treatment [35]. Although it was not the primary objective of this study, it is important to mention the serious implications of BVDV on bovine reproduction, including infertility, abortions, and congenital malformations [21]. In this context, previous studies have reported a predominant presence of BVDV subtype 1b in aborted fetuses [16], which is consistent with the findings of our study.

The combined use of techniques (VI, RT-nPCR, and LI) proved effective in confirming cases. However, the low sensitivity of some tests and the genetic variability of the virus may limit detection [8]. Therefore, it is important to emphasize that using a combination of diagnostic methods can enhance the likelihood of successful detection. Argentina exhibits a high level of BVDV circulation, particularly within extensive beef cattle production systems, which dominate the national livestock sector [10,11]. These extensive conditions likely hinder the early detection and removal of PI animals, an essential component in controlling viral distribution. In this context, the experience of countries like Ireland, where a national BVDV eradication program has been in place since 2013, demonstrates that coordinated strategies, supported by legislation, centralized data systems, and stakeholder engagement, can significantly reduce the prevalence of PI animals and the economic burden of the disease [36,37]. Implementing similar approaches in Argentina, adapted to its specific production conditions, could represent a valuable step toward mitigating the economic impact of BVDV in the future.

## 5. Conclusions

This retrospective study provides a comprehensive overview of postnatal BVDV outbreaks in cattle from Central Argentina, highlighting the endemic nature of the disease despite decades of awareness and available vaccines. The findings emphasize the clinical and pathological variability of BVDV infections, the predominance of subtype 1b, and the challenges posed by extensive production systems. The lack of significant differences between AI and MD in terms of morbidity and mortality underscores the complexity of disease expression and the limitations of clinical diagnosis. These results reinforce the urgent need for integrated control strategies, including improved vaccination protocols, enhanced diagnostic surveillance, and systematic identification and removal of PI animals. Addressing these challenges is essential for reducing the economic burden of BVDV and improve the health and productivity of cattle herds in Argentina.

## Figures and Tables

**Table 1:** Main clinical signs in 43 outbreaks associated with acute infection (AI) or mucosal disease (MD) due to Bovine Viral Diarrhea Virus (1995–2024).

	Acute Infection (*n* = 17)		Mucosal Disease (*n* = 26)	
	*n*	%	*n*	%
Weakness	6	35.29%	18	69.23%
Sialorrhea	11	64.71%	14	53.85%
Emaciation	8	47.06%	12	46.15%
Diarrhea	7	41.18%	10	38.46%
Nasal discharge	8	47.06%	8	30.77%
Dehydration	2	11.76%	9	34.62%
Fever	8	47.06%	4	15.38%
Dry muzzle	3	17.65%	6	23.08%
Dyspnea	2	11.76%	5	19.23%
Epiphora	5	29.41%	2	7.69%
Submandibular edema	-	-	1	3.85%

**Table 2:** Main gross findings in the 53 autopsies associated with acute infection (AI) or mucosal disease (MD) due to Bovine Viral Diarrhea Virus (1995–2024).

	Acute Infection (*n* = 20)		Mucosal Disease (*n* = 33)	
	*n*	%	*n*	%
Oral cavity				
lips	0	0.0%	9	27.27%
gum	7	35.00%	17	51.52%
cheek	6	30.00%	8	24.24%
tongue	6	30.00%	20	60.61%
hard palate	3	15.00%	13	39.39%
soft palate	1	5.00%	4	12.12%
Esophagus	15	75.00%	24	72.73%
Forestomachs				
reticulum	0	0.0%	1	3.03%
rumen	2	10.00%	11	33.33%
omasum	0	0.0%	2	6.06%
Abomasum	10	50.00%	19	57.58%
Small intestine				
duodenum	0	0.0%	0	0.0%
jejunum	2	10.00%	0	0.0%
ileum	4	20.00%	19	57.58%
Large intestine				
colon	2	10.00%	7	21.21%
cecum	0	0.00%	3	9.09%
rectum	0	0.00%	2	6.06%
Skin	11	55.00%	18	54.55%
Lymph nodes	6	30.00%	10	30.30%

**Table 3:** Histopathologic findings in the 48 autopsies associated with acute infection (AI) or mucosal disease (MD) due to Bovine Viral Diarrhea Virus (1995–2024).

	Acute Infection (*n* = 16)		Mucosal Disease (*n* = 32)	
	*n*	%	*n*	%
Cheek—multiple erosions	2	12.50%	2	6.25%
Tongue				
ulcerative glossitis	3	18.75%	11	34.38%
erosive glossitis	1	6.25%	3	9.38%
glossitis	4	25.00%	9	28.13%
Larynx—erosive laryngitis	0	0.00%	1	3.13%
Glottis—erosive glottitis	0	0.00%	1	3.13%
Esophagus				
erosive esophagitis	2	12.50%	5	15.63%
ulcerative esophagitis	3	18.75%	9	28.13%
esophagitis	2	12.50%	13	40.63%
Rumen				
erosive ruminitis	5	31.25%	6	18.75%
ulcerative ruminitis	0	0.00%	4	12.50%
ruminitis	3	18.75%	7	21.88%
Abomasum				
erosive abomasitis	0	0.00%	1	3.13%
ulcerative abomasitis	2	12.50%	5	15.63%
abomasitis	8	50.00%	9	28.13%
Small intestine				
erosive enteritis	3	18.75%	8	25.00%
ulcerative enteritis	3	18.75%	11	34.38%
enteritis	11	68.75%	24	75.00%
dilation of glands	6	37.50%	17	53.13%
Peyer’s patch necrosis	1	6.25%	7	21.88%
Large intestine				
erosive enteritis	1	6.25%	7	21.88%
ulcerative enteritis	1	6.25%	2	6.25%
enteritis	1	6.25%	7	21.88%
dilation of glands	1	6.25%	5	15.63%
Skin				
necrosis of the stratum corneum	4	25.00%	3	9.38%
dermatitis	5	31.25%	5	15.63%
follicular depletion	0	0.00%	3	9.38%
Spleen				
lymphoid depletion	3	18.75%	14	43.75%
splenitis	2	12.50%	2	6.25%
hemosiderin	4	25.00%	2	6.25%
Lymph nodes				
lymphoid depletion	2	12.50%	7	21.88%
lymphadenitis	4	25.00%	9	28.13%
Liver—portal hepatitis	4	25.00%	12	37.50%
Lung				
bronchopneumonia	2	12.50%	2	6.25%
interstitial pneumonia	12	50.00%	16	50.00%
emphysema	7	43.75%	8	25.00%
Kidney—interstitial nephritis	7	43.75%	8	25.00%
Central Nervous System				
gliosis	4	25.00%	6	18.75%
meningitis	1	6.25%	1	3.13%
Purkinje cell dysgenesis	1	6.25%	0	0.00%
Heart—endocarditis, myocarditis and epicarditis	4	11.54%	3	9.38%
Adrenal gland adrenalitis	0	0.00%	2	6.25%

**Table 4:** Diagnostic techniques and characterization of bovine viral diarrhea virus (BVDV) in the 50 outbreaks analyzed using different diagnostic techniques.

Outbreak	Viral Isolation	RT-nPCR ^1^	LI ^2^	Biotype ^3^	Subtype
1	+	+	NA	ncp	2a
2	+	+	NA	cp	1b
3	+	+	NA	ncp	2b
4	+	+	NA	cp	2b
5	+	+	NA	cp	1b
6	+	NA	NA	cp	-
7	+	+	NA	cp	1a
8	+	NA	NA	cp	-
9	+	NA	NA	ncp	-
10	+	+	NA	cp	1b
11	+	+	NA	ncp	1b
12	+	+	NA	ncp	1b
13	+	+	NA	ncp	1b
14	+	+	NA	cp	1b
15	+	+	NA	cp	1b
16	+	+	NA	cp	1b
17	+	NA	NA	ncp	-
18	+	+	NA	cp	1b
19	+	+	NA	ncp/cp	1b
20	+	+	NA	cp	1b
21	+	+	NA	ncp/cp	1i
22	+	+	NA	cp	1b
23	+	+	NA	ncp	2b
24	-	+	NA	-	1a
25	-	+	NA	-	1b
26	+	+	NA	cp	1a
27	+	+	NA	ncp/cp	1a
28	-	+	NA	-	1b
29	+	+	NA	ncp/cp	1a
30	+	NA	NA	ncp	-
31	+	+	NA	ncp	1b
32	+	NA	NA	cp	-
33	+	+	NA	ncp/cp	2b
34	+	+	NA	ncp	-
35	+	+	NA	ncp	1b
36	+	+	NA	ncp	1b
37	+	+	+	ncp	1b
38	+	+	+	cp	1a
39	+	+	+	cp	-
40	+	+	NA	ncp	1
41	+	+	NA	ncp/cp	-
42	-	NA	+	-	-
43	-	NA	+	-	-
44	+	+	+	ncp/cp	-
45	+	+	+	ncp	1
46	-	+	NA	-	-
47	+	+	NA	ncp/cp	-
48	-	+	+	-	-
49	+	+	+	ncp	-
50	+	+	NA	cp	-

^1^ RT-nPCR = Nested RT-PCR; ^2^ LI = lateral immunochromatography; NA = Not analyzed; ^3^ Biotype: ncp = non-cytopathic; cp = cytopathic.

## Data Availability

Data will be available upon request.

## Supplementary Materials

The following supporting information can be downloaded at https://www.mdpi.com/article/10.3390/v17101359/s1, Table S1. Epidemiological information of 50 postnatal outbreaks of disease associated with Bovine viral diarrhea virus infection in cattle in Central Argentina.

## Abbreviations

The following abbreviations are used in this manuscript:

BVDV	Bovine viral diarrhea virus
AI	acute infection
MD	mucosal disease
cp	cytopathic
ncp	non-cytopathic
PI	persistently infected
LI	lateral immunochromatography
