# Supplementary material for: Retrospective Analysis of 50 Postnatal BVDV Outbreaks in Cattle from Central Argentina: Clinical, Pathological, and Epidemiological Insights
Source: Viruses. 2025 Oct 11;17(10):1359. doi: 10.3390/v17101359 (PMC12567612; doi:10.3390/v17101359)
Supplement: Supplementary file 1 [file viruses-17-01359-s001.zip › viruses-3874016-supplementary.pdf]

natal outbreaks of disease associated with Bovine viral diarrhea virus infection in cattle in Central Argentina.

epartment	Month-Year	Categories affected	Type of presentation	Production system
s/Lobería	March-1995	Steers/Heifers	AI	Breeding
/Necochea	September-1995	Heifers	MD	Wintering
/Necochea	April-1998	Steers	AI	Breeding
/Ayacucho	December-1998	Steers	MD	Breeding
s/Balcarce	March-1999	Heifers	MD	Breeding
s/Laprida	December-2002	Calves	MD	Breeding
nque Lauquen	May-2003	Calves	MD	Feedlot
eral López	March-2004	Steers/Heifers/Calves	MD	Breeding
s/Laprida	March-2004	Calves	AI	Breeding
oronel Suarez	March-2004	Steers	MD	Breeding
neral Alvarado	March-2008	Steers	AI	Wintering
/9 de Julio	April-2008	Steers	AI	Breeding
s/Lincoln	November-2008	Steers	AI	Feedlot
neral Lamadrid	March-2009	Calves	MD	Breeding
res/Azul	April-2009	Calves	MD	Wintering
es/Bolivar	June-2009	Yearling bulls	MD	Breeding
oronel Suarez	November-2009	Steers/Heifers	AI	Breeding
s/Laprida	February-2010	Calves	MD	Breeding
25 de Mayo	September-2010	Steers/Heifers	MD	Feedlot
/Las Flores	October-2010	Steers	MD	Bull breeding farms
/Ayacucho	December-2010	Heifers	MD	Breeding
eral López	September-2011	Heifers	MD	Breeding
Magdalena	March-2013	Yearling bulls/Heifers	AI	Bull breeding farms
s/Saladillo	April-2013	Steers	-	Breeding
/Las Flores	May-2013	Calves/Steers	-	Breeding
eneral Alvear	July-2013	Steers	MD	Breeding
eneral Alvear	July-2013	Steers	MD	Breeding
es/Bolivar	September-2013	Calves	-	Breeding
Mar Chiquita	December-2013	Heifers	MD	Breeding
Tres Arroyos	January-2014	Calves	AI	Breeding
s/Pehuajó	February-2014	Calves	AI	Breeding
/Necochea	August-2014	Steers/Heifers	MD	Breeding
s/Saladillo	October-2014	Steers/Heifers	MD	Breeding
an Cayetano	July-2015	Calves	AI	Breeding
ronel Dorrego	April-2016	Calves	AI	Breeding
eral López	May-2016	Calves	AI	Breeding
nque Lauquen	April-2017	Bulls/Calves	AI	Breeding
25 de Mayo	August-2018	Calves	MD	Breeding
es/Rauch	August-2018	Steers/Heifers	MD	Breeding
s/Balcarce	October-2018	Steers	AI	Breeding
s/Balcarce	February-2019	Calves	MD	Dairy farm
ronel Pringles	January-2020	Steers	-	Breeding
/Olavarría	February-2021	Steers	-	Breeding
en de Patagones	February-2022	Steers	MD	Breeding

neral Lamadrid	August-2022	Steers	AI	Breeding
es/Maipú	November-2022	Steers	-	Breeding
Tres Arroyos	November-2022	Calves	MD	Breeding
General Pinto	June-2023	Calves	-	Feedlot
neral Alvarado	March-2024	Calves	AI	Breeding
/Ayacucho	June-2024	Calves	MD	Breeding

AI: Acute infection; MD: Mucosal diseases
